# Effect of Zinc and Copper Nanoparticles on Drought Resistance of Wheat Seedlings

**DOI:** 10.1186/s11671-017-1839-9

**Published:** 2017-01-19

**Authors:** Nataliya Taran, Volodymyr Storozhenko, Nataliia Svietlova, Ludmila Batsmanova, Viktor Shvartau, Mariia Kovalenko

**Affiliations:** 1 Educational and Scientific Centre ‘Institute of Biology and Medicine’, Taras Shevchenko National University of Kyiv, 64/13, Volodymyrska Street, Kyiv, 01601 Ukraine; 2 Institute of Plant Physiology and Genetics, National Academy of Sciences of Ukraine, 31/17, Vasylkivska St., Kyiv, 03022 Ukraine

**Keywords:** Cu,Zn-nanoparticles, Wheat, Seed treatment, TBARS, Superoxide dismutase, Catalase

## Abstract

The effect of a colloidal solution of Cu,Zn-nanoparticles on pro-oxidative/antioxidative balance and content of photosynthetic pigments and leaf area of winter wheat plants of steppe (Acveduc) and forest-steppe (Stolichna) ecotypes was investigated in drought conditions. It has been shown that Cu,Zn-nanoparticles decreased the negative effect of drought action upon plants of steppe ecotype Acveduc. In particular, increased activity of antioxidative enzymes reduced the level of accumulation of thiobarbituric acid reactive substances (TBARS) and stabilized the content of photosynthetic pigments and increased relative water content in leaves. Colloidal solution of Cu,Zn-nanoparticles had less significant influence on these indexes in seedlings of the Stolichna variety under drought.

## Background

Climate changes are most often associated now with global warming and droughts. It is supposed that about 1.8 billion people will be faced with absolute water shortage in the first quarter of the twenty-first century and 65% of the human population will live in conditions of partial shortage of water taking into account modern forecasts [1].

Grain cereals formed more than 50% of the general crop on a world scale, and use of their seeds is of paramount importance for production of food and industrial raw materials [2].

At the same time, grain crops strongly suffer from insufficient water supply, showing various morphological, physiological, biochemical, and molecular responses to drought. Water deficit can cause negative reversible and irreversible physiological changes of plant state in the vegetative and reproductive periods of plant development [3–6].

The modern agricultural technologies aimed at increasing productivity often do not take into account the environmental factor, especially in developing countries.

At the same time, the cost of fertilizers and irrigation increases in connection with the rising cost of primary resources every year, needed for their implementation in agricultural production, which threatens food security.

In this regard, to address the issue of increase of productivity and sustainable environmental management of agriculture in drought conditions, new environmentally friendly approaches that do not require large expenditures are needed. They should be based on promoting the adaptation of plant capacity in drought conditions [7].

The use of nanotechnology, we believe, can help in solving this issue. The relative cheapness of production of nanoparticles can serve it [8], as well as the low consumption of these preparations upon the crop area [9] and the low level of phytotoxicity.

The influence of nanoparticles on physiological state of plants at the different levels of their organization, beginning from molecular, has been studied at various plants. It is known that nanoparticles in different concentrations can impact both positive and negative biological effects [10]. In a number of papers were described the toxic effects of nanoparticles on plant growth, their development, and reproduction [11–14].

The results of our previous works have shown that nanoparticles received by physical synthesis are less toxic [15] in comparison with nanoparticles received by chemical synthesis.

The use of binary compositions of nanoparticles in agro-technologies to increase the biological productivity of agricultures in connection with its byway causes special interest for us.

In particular, the most actual is to find ways to increase the adaptation potential of cultivated plants with the use of nanopreparations in stressful conditions.

In this connection, there are interesting results obtained with plant agricultures, particularly winter wheat grown in conditions of water deficit in the present experiment.

The aim of the work was the estimation of Cu,Zn-nanoparticle colloidal solution influence upon pro-oxidative/antioxidative balance and photosynthetic and morphometric indexes of wheat seedlings of forest-steppe and steppe ecotypes under drought.

## Methods

Winter wheat varieties of forest-steppe (Stolichna) and steppe (Acveduc) ecotypes were used.

Seed treatment was carried out with colloidal solution of Cu and Zn nanoparticles (NPs). Variants of the experiment were as follows: (1) control (grown in optimum conditions of water supply); (2) seed pre-treatment with colloidal solution of Cu and Zn nanoparticles, grown in optimum conditions of water supply; (3) drought; and (4) drought + seed treatment with colloidal solution of NPs.

Colloidal solutions of metal nanoparticles were developed by the Department of the Technology of Structural Materials and Material Science of the National University of Life and Environmental Sciences of Ukraine and obtained as a result of dispersing copper and zinc granules by impulses of electric current with amplitude of 100–2000 A [16]. Seeds were pre-soaked in experimental mixtures (1 part of mother colloid solution to 100 parts of water) for 4 h and then washed with distilled water and placed in a thermostat for 1 day at 25 °C. The control variant was pre-soaked in distilled water.

Plants were grown in sand culture at 25 °C and watered with distilled water (photoperiod 16 h, irradiance of 250 μmol m^2^ s^−1^). Humidity of the soil was maintained at the level of 70% of total moisture capacity [17]. Plant density was 70 plants in one pot (0.29 m^2^). The drought was initiated on the eighth day after emergence of seedlings by termination of watering and support at level of 30% of total moisture capacity for 3 days. Then the physiological and morphometric indexes of seedlings were measured in the leaves of all levels.

The level of lipid peroxidation was evaluated by accumulation of thiobarbituric acid reactive substances (TBARS), using the reaction with 2-thiobarbituric acid [18]. The activity of superoxide dismutase (SOD, EC 1.15.1.1) was determined according to Giannopolitis and Ries [19], and the activity of catalase (CAT, EC 1.11.1.6) was determined according to Kumar and Knowles [20].

The content of photosynthetic pigments in leaves was determined after their extraction in dimethyl sulfoxide at 65 °C for 3 h [21].

The relative water content (RWC) in leaves was determined according to Smart and Bingham [22] and leaf area (LA) according to Chanda and Singh [23].

The experiment was conducted with at minimum three biological and analytical replications. The data analysis was performed using Microsoft Office Excel and Student’s *t* test at significance level *p* ≤ 0.05.

## Results and Discussion

Based on the results of our previous experiments with perennial water plant *Pistia stratiotes* L. (Araceae), a colloidal solution of Cu,Zn-nanoparticles caused a negative synergistic effect on amino acid composition of plants with a reduction of total content of amino acids (by 15%) and individual amino acids more than two times. At the same time, it caused significant changes of the morphometric indexes in plants [10].

**Fig. 1:**
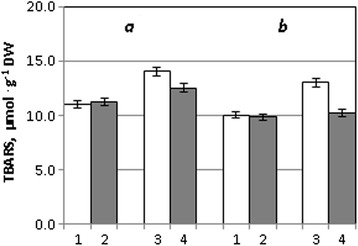
The content of TBARS in leaves of winter wheat seedlings of the Stolichna (*a*) and Acveduc (*b*) varieties after seed treatment with NPs and drought: *1* control, *2* NPs, *3* drought, and *4* NPs + drought

Analysis of the results of this work show that cultivation of wheat seedlings at drought conditions led to an increase in TBARS content in photosynthetic tissues both in Stolichna (by 27%) and the Acveduc (by 30%) varieties, which demonstrate the development of oxidative stress. At the same time, it should be noted that seed treatment by metal nanoparticles did not cause the increase of TBARS content in optimum conditions of seedling growth but drought caused the decrease of their content in leaves of Stolichna (by 11%) and Acveduc (by 22%) varieties (Fig. 1).

SOD activity in the leaves of seedlings of the forest-steppe ecotype variety (Stolichna) was higher than that in the leaves of the steppe ecotype variety (Acveduc) by 31% at normal conditions.

**Fig. 2:**
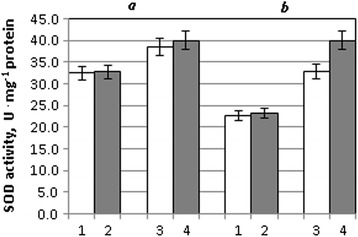
SOD activity in leaves of wheat seedlings of the Stolichna (*a*) and Acveduc (*b*) varieties after seed treatment with NPs and drought: *1* control, *2* NPs, *3* drought, and *4* NPs + drought

It also emerged that seed treatment with colloidal solution of Cu,Zn-nanoparticles caused the increase of SOD activity by 22% in drought conditions in the leaves of seedlings of the steppe ecotype variety Acveduc compared with seedlings of this variety, which were grown in drought conditions without seed treatment with NPs (Fig. 2).

**Fig. 3:**
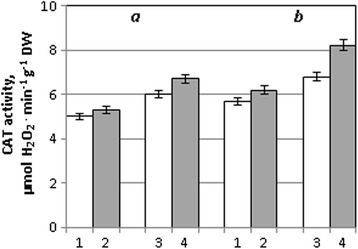
CAT activity in leaves of winter wheat seedlings of the Stolichna (*a*) and Acveduc (*b*) varieties after seed treatment with NPs and drought: *1* control, *2* NPs, *3* drought, and *4* NPs + drought

A similar trend was observed for catalase. The activity of this enzyme was unchanged after seed treatment with NPs. At the same time, metal nanoparticles induced the increase of catalase activity in leaves of the Acveduc variety by 21% under drought in comparison with seedlings, which were in drought conditions without pre-treatment of seeds with NPs (Fig. 3).

Based on our hypothesis, activation of the antioxidative system of plants under the influence of NPs’ binary composition is associated with the possible involvement of nanoparticles to enzymatic reactions of plant metabolism.

This hypothesis is confirmed by the fact that NPs can be transported through epidermal cells, and owing to their small size, they can contact with the high-molecular organic compounds of cells. At the same time, it has proved possible the intercellular transport of nanoparticles through the plasmodesmata [24].

In our opinion, the decrease in TBARS accumulation in leaves in our experiment under drought after pre-treatment of seeds is associated with increased activity of the antioxidative enzymes SOD and CAT. In connection with the increased activity of SOD and CAT, the level of TBARS decreased.

The content of photosynthetic pigments in the leaves of winter wheat seedlings of forest-steppe (Stolichna) and steppe (Acveduc) varieties also differ both in the drought conditions after pre-treatment of seedlings with the colloidal solution of nanoparticles and in those without this treatment.

The content of total chlorophyll (Chl) in leaves of the Acveduc variety was higher by 13% in comparison with that of the Stolichna variety at normal conditions.

After seed treatment with the colloidal solution of NPs, a 13% increase of the chlorophyll content in leaves of the Stolichna variety in comparison with the control was observed, while no significant difference in leaves of the Acveduc variety was observed.

Drought at 30% of the total moisture capacity induced a significant decrease in the total content of chlorophyll in the leaves of seedlings of both varieties Stolichna and Acveduc, and it was more significant for the variety Acveduc (45%) compared with the variety Stolichna (29%).

**Fig. 4:**
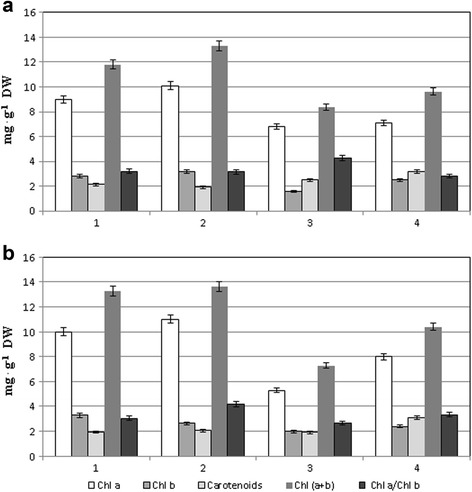
The content of photosynthetic pigments in leaves of winter wheat varieties Stolichna (**a**) and Acveduc (**b**) after seed treatment with NPs and drought: *1* control, *2* NPs, *3* drought, and 4–NPs + drought

A less significant reduction of chlorophyll content was observed in the leaves of both varieties of wheat after seed treatment during drought action than without treatment. In particular, chlorophyll content decreased with treatment by 18 and 22%, respectively, under drought compared to the control (Fig. 4).

In addition, changes in content of two types of chlorophyll and their ratio for the individual variants were observed. Seed treatment with colloidal solution of NPs induced increase of Chl *a* content both in the leaves of the Stolichna (by 12%) and Acveduc (by 10%) varieties. Content of Chl *a* in leaves of the Stolichna variety in drought conditions decreased by 24%; however, seed treatment with NPs induced the decrease of Chl *a* content by 21% under drought. For the Acveduc variety, we observed a similar trend. Drought without NPs caused a decrease of Chl *a* content by 47%, although seed treatment with colloidal solution of NPs under drought decreased the reduction of Chl *a* by 20% compared with the control (Fig. 4).

Chl *b* content also varied in drought conditions without NP treatment and with it. The increase of Chl *b* (by 14%) in the leaves of the Stolichna variety was observed, but it was reduced by 20% in the leaves of the Acveduc variety after seed treatment. At the same time, drought without NP treatment induced the decrease of Chl *b* content in leaves of the Stolichna (by 43%) and Acveduc (by 40%) varieties.

However, we observed the decrease of chlorophyll content in leaves of wheat seedlings of the Stolichna (by 11%) and Acveduc (by 27%) varieties after seed treatment by NPs under drought.

The Chl *a* to Chl *b* ratio in leaves of wheat seedlings changed under the combined action of NPs and drought. Changing of the Chl *a* to Chl *b* ratio in leaves of the Stolichna variety seedling after seed treatment with NPs was not observed. But in leaves of the Acveduc variety, this ratio increased by 37%. Drought induced increases of the Chl *a* to Chl *b* ratio by 32% in leaves of the Stolichna variety, but in leaves of the Acveduc variety, we observed the decrease of it by 18%.

Seed treatment with colloidal solution of NPs caused the decrease of the Chl *a* to Chl *b* ratio by 12% in leaves of the Stolichna variety seedlings and increase of it by 37% in leaves of the Acveduc variety seedlings. Drought, on the contrary, induced the increase of the Chl *a* to Chl *b* ratio by 32% in leaves of the forest-steppe variety Stolichna and the decrease of it by 12% in the steppe variety Acveduc (Fig. 3).

Changes in the content of photosynthetic pigments in leaves in response to nanopreparation influence were demonstrated by some authors [25, 26]. Interesting results were obtained with soybean plants with the addition of superparamagnetic iron oxide nanoparticles (SPIONs) in hydroponic culture. There was an increase in content of chlorophyll in the subapical leaves of soybean plants, although it did not lead to an increase in photosynthetic productivity. It was also noted that the action of SPIONs on chlorophyll content may have an impact on photochemical reactions [25].

Furthermore, the inclusions of nanoparticles in photosynthetic metabolism were confirmed. In particular, it was proved that the gold nanoparticles can be an artificial electron acceptor and a donor in photosynthesis [27].

The increase of the Chl *a* to Chl *b* ratio under nanoparticle action may indicate a change in the stoichiometry of light-harvesting complexes of photosystem I and photosystem II and, indirectly, a change of their activity in relation to each other [28].

The content of carotenoids in leaves of the Stolichna variety after influence of NPs was decreased by 10%. In the Acveduc variety, NPs had no effect on the content of carotenoids in leaves at a normal water supply. The content of total carotenoid under drought was decreased by 14% in leaves of the Stolichna variety, but in leaves of the Acveduc variety, it did not differ from the control. At the same time, after the seed treatment of NPs at drought, carotenoid content increased by 31% in leaves of the Stolichna variety and by 50% in leaves of the Acveduc variety.

In our work, the increase of carotenoid content in leaves of seedlings grown from seeds treated with solution of nanoparticles after drought action demonstrates the well-known adaptation mechanism [29].

In particular, the carotenoids are low-molecular antioxidants whose biosynthesis in leaves is increased in response to stress in order to quench reactive oxygen species on the one hand and to widen the absorption spectrum of available light radiation for plants due to physicochemical properties of their molecules on the other hand.

A significant increase of carotenoid content in seedlings of the drought-resistant variety, along with high activity of SOD and CAT in their leaves, indicates the higher antioxidative status of the plants investigated.

**Fig. 5:**
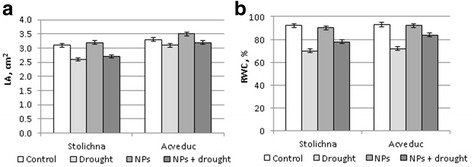
Leaf area (**a**) and relative water content (**b**) in winter wheat plants of the Stolichna and Acveduc varieties under NP treatment and drought

Seed treatment of wheat with colloidal solution of nanoparticles did not cause changes in LA under optimum water supply in both varieties. Drought induced the decrease of LA of the Stolichna (by 16%) and Acveduc (by 8%) seedlings. At the same time, drought induced a slight increase of LA in the drought-resistant variety Acveduc (by 5% compared to the previous variant) in nanoparticle-treated plants (Fig. 5).

The tendency to increase LA in plants treated with nanoparticles is described in the study of the effect of three different physical forms of zinc oxide nanoparticles upon morphometric parameters of maize plants. An increase of LA of the leaves of plants treated with all three forms individually was observed [30].

The RWC in leaves after seed treatment with colloidal solution of nanoparticles at normal water supply did not change in both varieties, although at drought conditions a decrease of this index in the Stolichna variety (by 24%) and the Acveduc variety (by 18%) was observed. In contrast, the combined effect of colloidal solution of nanoparticles and drought induced the increase of RWC by 8% in leaves of the Stolichna variety and by 10% in leaves of the Acveduc variety in comparison with the seedlings of both varieties exposed to the drought factor only (Fig. 5).

The positive impact on the physiological state of seedlings under drought conditions was found basing on the change of the relative water content in the leaves, which was higher in seedlings from seeds pretreated with a colloidal solution of NPs (Fig. 5). A similar effect was observed after exposure of wheat and maize plants to analcite nanoparticles [31].

Thus, the adaptive effect of Cu and Zn nanoparticles upon the state of photosynthetic apparatus in drought conditions was observed. It was manifested in the form of changes of the content and ratio of photosynthetic pigments, at the level of the antioxidant system, and ultimately at the organism level—in terms of the water regime of the seedlings and their morphometric parameters.

## Conclusions

In drought conditions, the colloidal solution of Cu,Zn-nanoparticles have a positive effect on pro-oxidative/antioxidative balance and morphometric indexes of leaves more in seedlings of the steppe ecotype (Acveduc) and less in seedlings of the forest-steppe ecotype (Stolichna). There was a decrease in TBARS accumulation and an increase of antioxidative enzyme (SOD and catalase) activity that characterize the increase of plant antioxidative status at the influence of nanoparticles in drought conditions.

Changing the ratio of chlorophyll in the leaves (Chl *a* to Chl *b*), along with a high content of carotenoids in the leaves, was a manifestation of plant adaptation to drought at the influence of the colloidal Cu and Zn nanoparticle solution.

In addition, changes in plant morphometric indexes such as leaf area and relative water content in leaves are the result of the adaptation mechanism induction of investigated plants at drought conditions.

## References

[1] Nezhadahmadi A, Prodhan ZH, Faruq G (2013) Drought tolerance in wheat. Sci World J 610721. doi: 10.1155/2013/610721. 10.1155/2013/610721 PMC3844267 24319376

[2] Mochida K, Shinozaki K (2013). Unlocking Triticeae genomics to sustainably feed the future. Plant Cell Physiol.

[3] Szegletes ZS, Erdei L, Tari I, Cseuz L (2000). Accumulation of osmoprotectants in wheat varieties of different drought tolerance. Cereal Res Commun.

[4] Zhu JK (2002). Salt and drought stress signal transduction in plants. Ann Rev Plant Biol.

[5] Lawlor DW, Cornic G (2002). Photosynthetic carbon assimilation and associated metabolism in relation to water deficits in higher plants. Plant Cell Environ.

[6] Yordanov I, Velikova V, Tsonev T (2000). Plant responses to drought, acclimation, and stress tolerance. Photosynthetica.

[7] Kang Y, Khan S, Ma X (2009). Climate change impacts on crop yield, crop water productivity and food security. Prog Nat Sci.

[8] Narayanan S, Sathy BN, Mony U, Koyakutty M, Nair SV, Menon D (2012). Biocompatible magnetite/gold nanohybrid contrast agents via green chemistry for MRI and CT bioimaging. ACS Appl Mater Interfaces.

[9] Batsmanova LM, Gonchar LM, Taran NY, Okanenko AA (2013). Using a colloidal solution of metal nanoparticles as micronutrient fertilizer for cereals. Proc Int Conf Nanomaterials Appl Properties.

[10] Olkhovych O, Volkogon M, Taran N, Batsmanova L, Kravchenko I (2016). The effect of copper and zinc nanoparticles on the growth parameters, contents of ascorbic acid, and qualitative composition of amino acids and acylcarnitines in *Pistia stratiotes* L. (Araceae). Nanoscale Res Lett.

[11] Oukarroum A, Barhoumi L, Pirastru L, Dewez D (2013). Silver nanoparticle toxicity effect on growth and cellular viability of the aquatic plant *Lemna gibba*. Environ Toxicol Chem.

[12] Chichiriccò G, Poma A (2015). Penetration and toxicity of nanomaterials in higher plants. Nanomaterials.

[13] Mustafa G, Komatsu S (2016). Toxicity of heavy metals and metal-containing nanoparticles on plants. Biochimica et Biophysica Acta.

[14] Cox A, Venkatachalam P, Sahi S, Sharma N (2016). Silver and titanium dioxide nanoparticle toxicity in plants: a review of current research. Plant Physiol Biochem.

[15] Konotop IO, Kovalenko MS, Ulynets VZ, Meleshko AO, Batsmanova LM, Taran NY (2014). Phytotoxicity of colloidal solutions of metal-containing nanoparticles. Cytol Genet.

[16] Lopatko KH, Aftandiliants EH, Kalenska SM, Tonkha OL. Mother colloidal solution of metals. B01J 13/00 Patent of Ukraine No. 38459 12 Jan 2009. http://uapatents.com/4-38459-matochnijj-kolodnijj-rozchin-metaliv.html.

[17] Grigoryuk IA, Tkachyov VI, Savinsky SV, Musienko NN (2003). Modern methods of investigations and estimation of drought tolerance and heat tolerance of plants: workbook.

[18] Andreyeva LI, Kozhemyakin LA, Kishkun AA (1988). Modification of method of lipid peroxides determination in the test with thiobarbituric acid. Lab Work.

[19] Giannopolitis CN, Ries SK (1977). Superoxide dismutases: I. Occurrence in higher plants. Plant Physiol.

[20] Kumar CN, Knowles N (1993). Changes in lipid peroxidation and lipolytic and free-radical scavenging enzyme during aging and sprouting of potato (Solanum tuberosum L.) seed-tubers. Plant Physiol.

[21] Wellburn AR (1994). The spectral determination of chlorophylls a and b as well, as the total carotenoids using various solvents with spectrophotometers of different resolution. J Plant Physiol.

[22] Smart RE, Bingham GE (1974). Rapid estimates of relative water content. Plant Physiol.

[23] Chanda SV, Singh YD (2002). Estimation of leaf area in wheat using linear measurements. Plant Breed Seed Sci.

[24] Zhai G, Walters KS, Peate DW, Alvarez PJJ, Schnoor JL (2014). Transport of gold nanoparticles through plasmodesmata and precipitation of gold ions in woody poplar. Environ Sci Technol Lett.

[25] Ghafariyan MH, Malakouti MJ, Dadpour MR, Stroeve P, Mahmoudi M (2013). Effects of magnetite nanoparticles on soybean chlorophyll. Environ Sci Technol.

[26] Falco WF, Queiroz AM, Fernandes J, Botero ER, Falcão EA, Guimarães FEG (2015). Interaction between chlorophyll and silver nanoparticles: a close analysis of chlorophyll fluorescence quenching. J Photochem Photobiol A Chem.

[27] Barazzouk S, Kamat PV, Hotchandani S (2005). Photoinduced electron transfer between chlorophyll a and gold nanoparticles. J Phys Chem B..

[28] Green BR, Durnford DG (1996). The chlorophyll-carotenoid proteins of oxygenic photosynthesis. Annu Rev Plant Physiol Plant Mol Biol.

[29] Havaux M, Niyogi KK (1999). The violaxanthin cycle protects plants from photooxidative damage by more than one mechanism. Proc Natl Acad Sci USA.

[30] Taheri M, Qarache HA, Qarache AA, Yoosefi M (2015). The effects of zinc-oxide nanoparticles on growth parameters of corn (SC704). STEM Fellowship J.

[31] Zaimenko NV, Didyk NP, Dzyuba OI, Zakrasov OV, Rositska NV, Viter AV (2014). Enhancement of drought resistance in wheat and corn by nanoparticles of natural mineral analcite. Ecologia Balkanica.

